# Recent research advances in the development of Dabie Banda virus vaccines

**DOI:** 10.1371/journal.pntd.0012411

**Published:** 2024-08-29

**Authors:** Chenyang Yu, Yuxiang Lin, Yixin Dai, Bingan Wu, Zhongtian Qi, Xijing Qian

**Affiliations:** 1 Department of Microbiology, Faculty of Naval Medicine, Naval Medical University, Shanghai, China; 2 College of Basic Medical Sciences, Naval Medical University, Shanghai, China; 3 Nursing Department, Faculty of Health and Wellness, Linxia Modern Career College, Gansu, China; WRAIR, UNITED STATES OF AMERICA

## Abstract

Severe fever with thrombocytopenia syndrome (SFTS) is a newly identified tick-borne viral hemorrhagic fever caused by Dabie Banda virus (DBV). The virus was first discovered in eastern China in 2009 and is now considered an infectious disease with a mortality rate ranging from 6.3% to 30%. The best strategy for controlling SFTS is to develop effective vaccines. However, no approved vaccines are currently available to prevent this disease, despite the number of extensive and in-depth studies conducted on DBV in the past few years. This review focuses on the structure of DBV and the induced host immune responses which are the fundamental factors in vaccine development, and thoroughly summarizes the current research progress on DBV vaccines. The developing DBV vaccines include protein subunit vaccines, live attenuated vaccines, recombinant virus vector vaccines, and DNA vaccines. At present, almost all candidate vaccines for DBV are in the laboratory development or preclinical stages. There remain challenges in successfully developing clinically approved DBV vaccines.

## Introduction

Severe fever with thrombocytopenia syndrome (SFTS) is a viral hemorrhagic fever caused by a novel tick-borne virus, Dabie Banda virus (DBV) which belongs to the bunyaviral family *Phenuiviridae* [1–3]. SFTS was first reported in eastern China in 2009, with a mortality rate ranging from 6.3% to 30% [4,5]. The main transmission vectors of this virus are ticks, and tick bites are the main routes causing human disease [6]. In addition, humans can also be infected through close contact with patients’ body fluids [7–10]. DBV infection manifests as fever, fatigue, myalgia, nausea, vomiting, and diarrhea. Laboratory tests often show thrombocytopenia, leukopenia, and elevated levels of several hepatic enzymes, indicating liver damage. In severe cases, fever, bleeding, multiple organ failure, or even death may occur, [11,12] especially in elderly patients. Age is a critical risk factor for SFTS disease prognosis, and notification and case fatality rates increase dramatically in patients over 50 years old [13,14].

The number of people infected with DBV significantly increased annually, and the mortality rate remained high [15]. It was listed as a priority pathogen by the World Health Organization in 2018 [16]. In recent years, researchers have conducted extensive and in-depth research on DBV, and its molecular structure, epidemiological characteristics, and pathogenic mechanisms have been clarified [11]. However, there remains no effective vaccine or drug available to prevent or treat DBV infection. Vaccination has always been the most effective method for preventing infectious diseases. For DBV, a relatively novel and highly pathogenic virus, the development of an affordable vaccine with optimal effectiveness and few adverse reactions is of particular importance. DBV vaccines can help to prevent infection or the onset of severe disease. This review provides a brief introduction to the structures of DBV and the immune responses induced by DBV infection. A comprehensive summary on the latest research progress on different types of DBV vaccines is also included.

## 1. SFTSV structures

**Fig 1:**
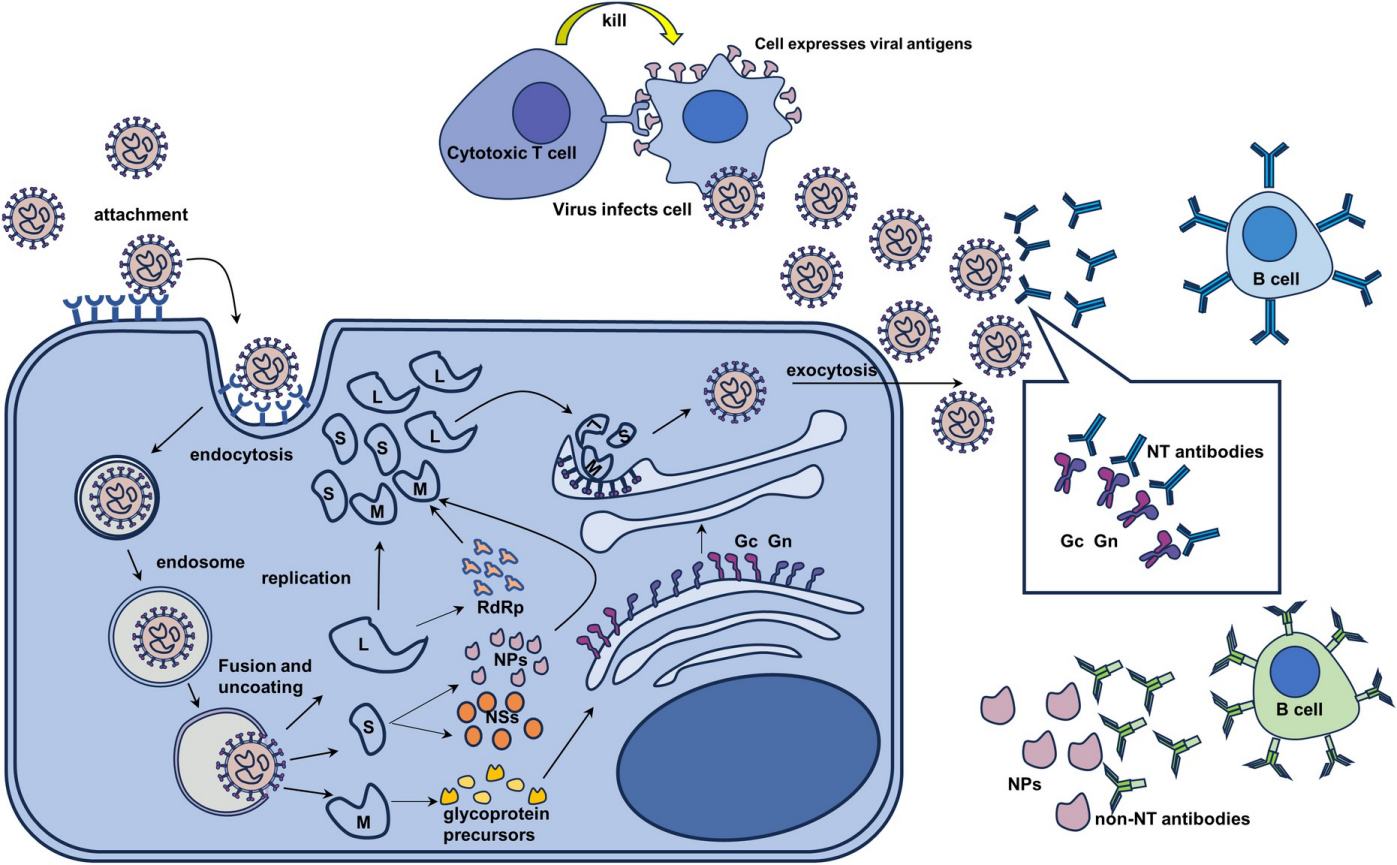
DBV life cycle and host immune responses. The attachment of the virus to host cell receptors and subsequent entry into the cell are facilitated by viral glycoproteins and mediated through clathrin-dependent endocytosis. The process of endocytosis leads to the formation of the endosomes that encapsulate the virus. As the pH value within the endosome decreases, it induces conformational changes in the glycoprotein structure on the viral surface, facilitating membrane fusion and subsequent release of the viral genome. RdRp and NPs are involved in viral RNA replication, followed with the transportation to Golgi complexes where they will interact with newly translated glycoproteins to commence assembly processes. The newly synthesized viruses are released from the Golgi apparatus to infect neighboring cells and trigger immune responses. Neutralizing antibodies secreted by B cells primarily bind to Gn and Gc, while non-neutralizing antibodies bind to Np. Additionally, cytotoxic T cells recognize and eliminate the infected cells.

DBV is a negative-sense RNA virus. Its genome consists of 3 fragments: large (L), medium (M), and small (S) [17]. The L-segment encodes RNA-dependent RNA polymerase (RdRp), which is responsible for mediating viral RNA synthesis and replication. The M-segment encodes glycoprotein precursors, which are cleaved into glycoprotein N (Gn) and glycoprotein C (Gc) by host proteases (Fig 1) [18]. Gn and Gc of DBV are 2 major antigenic components on the viral surface and proposed to be membrane fusion proteins critical for DBV infection [19]. More importantly, Gn and Gc are the targets of specific neutralizing antibodies [20]. Previous studies have identified DBV-neutralizing antibody epitopes on the head region of Gn, indicating that Gn and Gc are promising antigens for vaccine development [19,21,22]. Gn has 3 subdomains (I, II, and III), with N-linked glycans at residues 33 and 63 in subdomain I, which are the targets of neutralizing antibodies. Additionally, Gn also mediates viral attachment to specific receptors on host cells. Gc is a class II fusion protein responsible for mediating membrane fusion [23]. During the fusion process between DBV and the cell membrane, Gc undergoes a pH-dependent conformational rearrangement, resulting in the transition from the prefusion dimer to the post fusion trimer, exposing the fusion ring to induce membrane fusion [24]. The S segment encodes viral nucleoprotein (NP) and nonstructural proteins (NSs) (Fig 1). NPs encapsulate viral genomic RNA, forming a ribonucleoprotein (RNP) to maintain the stability of the viral RNA. NSs are important virulence factors for DBV and can inhibit the type I interferon (IFN) signaling pathway, assisting the virus in evading host immune responses. Furthermore, NSs play important roles in the virus replication process [25–27].

## 2. Host immune responses against SFTSV

### 2.1 Innate immune responses

When the host body is infected by SFTSV, a series of immune responses are triggered. During the early stage of DBV infection, the mononuclear phagocytic system, which consists of monocytes, macrophages, and dendritic cells (DCs), is the primary target. The number of monocytes decreases and the monocytes become dysfunctional at the early stage of DBV infection, while macrophages are the main target cells at the late stage of DBV infection. Moreover, myeloid dendritic cells (mDCs) may be deficient in maturation and antigen presentation, resulting in the inability to activate specific humoral responses. The role of natural killer (NK) cells in DBV infection remains to be determined. Nevertheless, one study found that increased NK cells are associated with severe disease conditions [17]. In another study, NK cells significantly decreased during the first week of DBV infection and then quickly returned to normal levels. Interferon plays a number of roles in both innate and acquired immunity. It has powerful and broad-spectrum antiviral activity [28,29]. It was recently discovered that there is a much greater incidence of overactive IFN-1 pathways and elevated ISG expression in fatal cases of SFTS. There is also a strong link between high ISG expression and more severe symptoms. Therefore, it appears that an increase in IFN-1 might cause more harm than may be beneficial [30].

### 2.2 Adaptive immune responses

Adaptive immune responses include humoral and cellular immunity. Humoral immunity is primarily mediated by the production of specific antibodies, which are immunoglobulins secreted by plasma cells of B cell origin. Specific antibodies targeting DBV can be detected approximately 1 week after the onset of the disease [31], of which specific IgG usually occurs 2 to 9 weeks after onset, increases to its peak level after 6 months and remains at detectable levels for at least 4 years [32,33]. Serum-specific IgM synthesis occurs 4 to 21 days after infection, reaches its peak after 4 weeks, and significantly decreases thereafter [15,34]. Guo and colleagues has shown that DBV monoclonal antibodies with neutralizing activity can specifically recognize and bind to the a6 helix in Gn subdomain III of DBV, which could be an ideal target for DBV vaccines [21]. Non-neutralizing antibodies mainly recognize and bind to DBV NPs [22,35–37] (Fig 1). Lee and colleagues revealed that NPs have high immunogenicity, and many monoclonal antibodies (such as H2A12, H2A4, H2E4, H2F4, and H1C2) can recognize linear and conformational epitopes of DBV NPs, but NP-specific antibodies have no significant neutralizing activity [38]. In addition, antibodies targeting NPs are usually detectable in the early stages of infection, making NPs a target antigen for the early diagnosis of DBV infection [39]. Although non-neutralizing antibodies are sometimes beneficial for promoting viral entry and replication, which is called antibody-dependent enhancement (ADE), no study concerning ADE in SFTSV infection has been conducted until now. Cellular immunity is mediated by CD4+ and CD8+ T lymphocytes. Notably, the number of CD3+, CD4+, and CD8+ T cells in severe patients was lower than that in mild patients [40], and the number of Th1, Th2, and Treg cells was significantly lower in patients who died than in other patients. This suggests that the severity of DBV infection is related to the activation of immune function. Furthermore, increased HLA-DR and CTLA4 might predict effective infection control and recovery in patients [41]. Generally, decreased counts, abnormal proportions, and dysfunctions of T lymphocyte subsets contribute greatly to DBV infection. Therefore, immune homeostasis is an important factor that needs to be considered during vaccine development.

## 3. Research progress on DBV vaccines

### 3.1 Protein subunit vaccines

Protein subunit vaccines are usually composed of viral proteins that can elicit robust immune responses against DBV and are among the safest vaccine types. The efficacy of subunit vaccines is relatively low, and they need to be used in combination with adjuvants to exert good immune protective effects (Table 1). Although there are currently few reports suggesting that fully inactivated DBVs or DBV protein subunits can be used as candidate vaccines, Kim and colleagues evaluated the effect of a protein subunit vaccine with 24 monomer subunit self-assembling ferritin (FT) nanoparticles on the head region of DBV Gn (GnH) [42]. Ferritin nanoparticles, which is an outstanding vaccine carrier platform for inducing strong protective immunity against infectious agents, and that GnH-FT nanoparticles can be purified by anion exchange chromatography and size exclusion chromatography. Furthermore, they demonstrated the immunogenicity of GnH-FT nanoparticles, and 8- to 10-week-old BALB/c mice were intramuscularly injected with a total of 3 doses of GnH-FT nanoparticles at 3-week intervals (1 μg, 5 μg, or 10 μg) combined with adjuvants (MF59, AddaVax). The results showed that a 1 μg GnH-FT dose was sufficient for inducing robust humoral immunity and cellular immunity in the host body and fully protected BALB/c mice from DBV challenge, which is consistent with the findings in aged ferrets. FTs are currently in the preclinical stage because of their prospective safety and outstanding protection efficacy, making them very promising vaccine candidates that deserve further clinical trials (Table 1). Moreover, the efficacy of DBV NSs as vaccine components has also been evaluated by some scholars [43]. However, preexposure vaccination with 100 μg of purified recombinant NSs expressed by *E*. *coli* with Freund’s complete adjuvant did not have a significant immune-protective effect on C57BL/6 mice (Table 1).

**Table 1:** The process of current vaccine development against DBV.

Vaccine type	Vaccine candidate	Advantages	limitations	References
Protein subunit vaccine	GnH-FT nanoparticles	Safety and high efficiency	High production costs, require multiple vaccinations	[42]
	DBV NSs subunit vaccines	Safety	Low efficiency	[43]
Live attenuated vaccine	rHB2912aaNSs rHB29NSsP102A	Low cost, high efficiency, and lasting protection	The genetic stability during in vivo passage remains to be studied	[46]
Recombinant vector vaccines	rVSV-based vaccine	Highly safe, induce broad-spectrum neutralizing antibodies	Cellular immune response is still unclear	[52]
	rVAC-based vaccine	Good immunogenicity and highly attenuated	Cellular immune response is still unclear	[63]
	rAd5-based vaccine	Safety, high immunogenicity, and tolerance	Cellular immune response is still unclear	[72]
	rAd5 Gn/Gn heterologous prime-boost strategy	High efficiency and wide range of application	Require twice vaccinations	[78]
DNA vaccines	pVax1-Gn pVax1-Gc pVax1-N pVax1-NSs and pVax1-RdRp	High efficiency	Require multiple vaccinations	[84]
	DBV recombinant plasmid DNA vaccine containing IL-12	Easy to develop	Insufficient immunogenicity, need effective adjuvants	[85]
mRNA vaccines	sGN-H and sGN-H-FT mRNA LNPs	High efficiency, safety, short production cycle, and low cost	Further study is needed to determine the duration and strength for potential long-term protection	[90]
	GP mRNA vaccine	High efficiency, safety	Technical limitations	[91]

### 3.2 Live attenuated vaccines

The manufacturing cost of live attenuated vaccines is low, and a single dose of vaccination is usually able to elicit a comprehensive and long-lasting immune response. Therefore, live attenuated vaccines are considered one of the most successful vaccines in history, with approximately 63% of vaccines approved by the Food and Drug Administration (FDA) being live attenuated [44]. By modifying the coding region of DBV (a wild-type derived recombinant Chinese isolate, HB29, genotype D) NS proteins, Yu and colleagues obtained 2 attenuated DBVs called rHB2912aaNSs and rHB29NSsP102A [45,46]. rHB2912aaNSs consists of a 12 amino acid short peptide sequence derived from the open reading frame (ORF) of NSs, which contains the first methionine and the last 11 amino acids of the NSs ORF [27]. rHB29NSsP102A has a single point mutation from proline to alanine at position 102 of the NSs coding sequence [45]. Researchers have found that aged ferrets (>4 years old) intramuscularly injected with rHB29NSsP102A (4 × 10^6^ PFUs) or rHB2912aaNSs (5 × 10^5^ PFUs) have lower viral loads in their serum and tissues and few symptoms, such as fever, thrombocytopenia, or infection-related deaths, indicating a high degree of weakened virus virulence. Nevertheless, these mutant viruses still induced a strong humoral immune response in the vaccinated ferrets and completely protected them from the fatal challenge of DBV (Table 1). Safety is an important indicator of live attenuated vaccines, since genetic drift may cause attenuated virus virulence to increase. Therefore, the application of live attenuated DBV vaccines might be limited especially in immunocompromised individuals. However, rHB2912aaNSs and rHB29NSsP102A did not appear to revert after serial passaging according to the study. Therefore, these 2 live attenuated vaccines have certain potential as candidate vaccines for DBV.

### 3.3 Recombinant virus vector vaccines

Viruses are natural high-quality carriers. During infection, viruses enter target cells, transmit key instructions to intracellular compartments, and drive efficient expression of target proteins. Therefore, using natural vector viruses with low pathogenicity to promote vaccine antigen expression is a promising strategy. Viral vector vaccines usually replace key genes of the vector virus by inserting target genes into the genome, reducing the virulence of the vector virus while retaining its immunogenicity and inducing specific immune responses against the target components in the host body. At present, the developed DBV vaccine virus vectors mainly include vesicular stomatitis virus (VSV), vaccinia virus (VAC), and adenovirus type 5 (Ad5).

#### 3.3.1 Recombinant vesicular stomatitis virus (rVSV)-based vaccine

VSV belongs to the vesicular virus genus, the family *Rhabdoviridae*, and is a membrane-bound negative-sense RNA virus [47,48]. Most VSV-infected individuals have no symptoms or only show flu-like symptoms, and can fully recover within a week [49]. In addition, because the natural hosts of VSV are livestock, humans usually lack immunity to VSV, and infection in humans can induce strong humoral and cellular immune responses [50]. The vector does not lose efficacy with repeated applications as it does not induce vector-specific humoral immunity [51]. Research has shown that the live attenuated rVSV-based vaccine candidate expressing DBV Gn/Gc glycoproteins (rVSV-DBV/AH12-GP) is highly safe and that 2 × 10^4^ PFU of rVSV-DBV/AH12-GP can elicit broad-spectrum neutralizing antibodies in interferon α/β receptor knockout (IFNAR-/-) C57/BL6 mice, completely protecting mice from lethal DBV challenge (Table 1), [52]. IFNAR-/- mice were vaccinated with rVSV-DBV/AH12-GP through different routes (intraperitoneal, intravenous, subcutaneous, or nasal), the protective effects of which were not significantly different. Because rVSV has been used as a vaccine carrier for various viruses [53], there may be antibodies targeting VSV in the human body. To determine whether these antibodies influence the effectiveness of the rVSV-DBV vaccine, researchers preimmunized mice with the rVSV vector and subsequently inoculated them with the rVSV-DBV vaccine. The results showed that the rVSV-DBV vaccine could still completely protect mice from high-dose lethal DBV challenge, indicating that human immune responses to the vector do not affect the protective effect of rVSV-DBV. In summary, rVSV-DBV is a promising candidate vaccine for DBV and has potential for further clinical development.

#### 3.3.2 Recombinant vaccinia virus (rVAC)-based vaccine

As one of the most successful vaccine vectors, vaccinia virus (VAC) has helped eliminate smallpox virus in humans [54,55], and VAC is one of the earliest viruses modified to express foreign genes [56–58]. LC16m8 (m8) and modified vaccinia Ankara (MVA) are third-generation VAC strains. These viruses retain immunogenicity, while their pathogenicity is comprehensively reduced [59,60]. Research has shown that m8 and MVA do not cause any severe complications and can enter target cells to activate their molecular life cycle, resulting in the expression of various viral and recombinant genes [60–62]. DBV vaccines based on m8 (m8-NP, m8-GPC, and m8-NP+GPC) have good immunogenicity and are highly attenuated (Table 1), [63]. m8-based DBV vaccines can express DBV nucleoprotein (NP), an envelope glycoprotein precursor (GPC) or a combination of these 2 viral proteins in target cells. Furthermore, cells infected with m8-GPC or m8-NP+GPC can produce DBV-like particles (VLPs), inducing robust immune responses. The experimental results indicated that 2 doses of m8-based DBV vaccines could elicit robust production of specific DBV antibodies in 8-week-old IFNAR-/- C57BL/6 mice and protect mice from lethal challenge with either 10^3^ TCID_50_ or 10^5^ TCID_50_ DBVs. According to the study, no pathological changes were observed in the livers, kidneys, spleens, or neck lymph nodes of mice inoculated with m8-GPC or m8-NP+GPC, while the mice in the control groups showed focal necrosis and inflammatory infiltration in these tissues. Passive serum transfer experiments showed that mouse serum inoculated with m8-GPC or m8-NP+GPC but not with m8-NP can protect naïve mice from lethal DBV attack. The above experimental results indicate that vaccination with the recombinant m8 strain that carries both GPs and DBV NPs can achieve satisfactory protective immunity against lethal attack by DBV in mice, and recombinant m8-NP+GPC is more likely to become a candidate vaccine. Because people born before 1980 were vaccinated against smallpox, there may be antibodies against VAC [64,65]. To determine whether they have an impact on the protective effect of the m8-based DBV vaccines, researchers preimmunized mice with the Lister vaccinia strain and inoculated them with the m8-based DBV vaccine [63]. The results suggested that preexisting VAC immunization does not significantly affect the protective immunity induced by the vaccine, although the role of cellular immunity in the protective immunity of m8-type DBV vaccines has not been clearly evaluated. However, the above findings are sufficient to validate m8-based DBV vaccines as promising DBV candidate vaccines worthy of further exploration.

#### 3.3.3 Recombinant replication-deficient human adenovirus 5 (rAd5)-based vaccine

Adenovirus is a widely distributed virus in nature, with approximately 52 types of human adenoviruses, of which types 5 and 2 are frequently used as vaccine carriers. Adenoviruses used as vaccine carriers knock out the genes required for replication, allowing them to infect human cells but not replicate [66,67]. rAd5 has been widely used in various human trials because of its high immunogenicity and tolerance to viruses such as SARS-CoV-2, Zika virus, dengue virus, and Ebola virus [68–71]. The recombinant human adenovirus type 5 vaccine expressing DBV Gn (Ad5-RABV-G-Gn) has been confirmed to have high immunogenicity and can induce the production of a high level of DBV neutralizing antibodies in 6- to 8-week-old C57BL/6 mice [72], 10^8^ GFU of Ad5-G-Gn is enough to protect them from lethal DBV challenges and significantly reduce viral loads in the spleen (Table 1). Therefore, Ad5-RABV-G-Gn is a promising candidate vaccine for DBV.

In recent years, a novel vaccination strategy involving priming with a recombinant virus vector and boosting with protein (a heterologous prime-boost strategy) has attracted tremendous attention. A number of studies have confirmed that this approach is more effective than the traditional homologous prime-boost strategy [73–77]. For example, Kim and colleagues reported that the rAd5 Gn/Gn heterologous prime-boost strategy formulated with an RNA adjuvant can elicit balanced Th1/Th2 immune responses and high titers of neutralizing antibodies in both C57BL/6 mice and nonhuman primates, activating robust humoral and T-cell-mediated responses. In addition, rAd5 Gn/Gn heterologous immunity can boost CD8+ and NK cells as well as macrophage/monocyte-related gene expression, and indiscriminately activate adaptive and innate immune pathways, which are crucial for the development of effective vaccines (Table 1) [78]. Since the majority of severe SFTS patients are elderly people, their immune responses are usually impaired due to the immune aging [79], and which may weaken vaccine efficacy. Therefore, Kim and colleagues conducted experiments on 16-month-old mice (elderly mice). The experimental results suggest that the heterologous prime-boost strategy can overcome the impact of immune aging on vaccine efficacy. Overall, a proper heterologous prime-boost strategy could be an optimal DBV vaccine strategy in the foreseeable future. Nevertheless, there remain potential adverse reactions that need to be investigated. The population may be universally immune to adenovirus infections; therefore, the titer of vector viruses in vaccine production is difficult to control. In addition, the pathogenicity and potential carcinogenic risk of viral vectors cannot be ignored, and further evaluation of their safety and efficacy is needed.

### 3.4 DNA vaccines

DNA vaccines typically consist of viral protective antigen genes and expression vectors. Plasmid DNA containing foreign antigens is usually controlled by a eukaryotic promoter, tail signal, and related enhancer gene units. The associated antigenic proteins can be expressed in various mammalian cells. Recombinant plasmids with foreign antigen-coding genes are directly introduced into human or animal cells by some method, and antigen proteins are synthesized in living immune cells through the transcription system of host cells, thus inducing immune responses [80,81]. In addition to stimulating-specific humoral and cellular immunity, they can also induce widespread immunity to multiple antigens, making them more suitable for developing vaccines targeting emerging pathogens [80,82]. Moreover, compared to traditional vaccines, DNA vaccines have the advantage of being relatively easy to develop, and the preparation technology for DNA vaccines is relatively mature; therefore, DNA vaccines can quickly respond to sudden infections caused by pathogens and are an important component of emergency vaccines [83].

Kwak and colleagues reported that DNA vaccines carrying DBV DNA plasmids (pVax1-Gn, pVax1-Gc, pVax1-N, pVax1-NSs, and pVax1-RdRp) can induce neutralizing antibodies and multifunctional-specific cellular responses against DBV in both 5- to 6-week-old BALB/c mice and more than 4-year-old ferrets (Table 1) [84]. Furthermore, elderly ferrets prone to developing fatal clinical signs, each containing 200 μg of pVax1-Gn, pVax1-Gc, pVax1-N, pVax1-NSs, and/or pVax1-RdRp in 200 μl of PBS, are completely protected from the fatal challenge of DBV via intradermal immunization with 3 doses at 2-week intervals. However, a single dose can only achieve partial protection. Further research suggested that the envelope glycoprotein Gn/Gc may be the most effective antigen for inducing protective immunity and that anti-envelope antibodies are crucial for immune protection. Kang and colleagues developed a recombinant plasmid DNA vaccine encoding the DBV envelope glycoproteins Gn and Gc, as well as NP-NS fusion antigens [85]. They fused the viral antigen with Fms-like tyrosine kinase 3 ligand (Flt3L) and incorporated the interleukin 12 (IL-12) gene into the plasmid to enhance cellular immunity. The findings of this research indicate that female IFNAR-/- C57BL/6 mice, aged 6 to 8 weeks and vaccinated with 4 μg of DNA vaccine containing IL-12 in the hind leg 3 times at 2-week intervals, can be completely protected from lethal DBV attacks. However, vaccines without IL-12 genes can only provide partial protection. In addition, enhanced T-cell responses specific to viral antigens were detected instead of neutralizing antibodies in immunized mice, suggesting that antigen-specific cellular immunity may play a major role in immune protection. Given that DNA vaccines have not induced sufficient immunogenicity in human trials compared to recombinant protein vaccines, further research is needed to develop optimal DBV vaccines involving the combination of DNA and proper target antigens, together with the incorporation of effective adjuvants (Table 1).

### 3.5 mRNA vaccines

mRNA vaccines are the third generation of nucleic acid vaccines. Compared with traditional vaccines, mRNA vaccines have many advantages, such as high efficiency, safety, short production cycles, and low cost. The 2023 Nobel Prize in Physiology or Medicine was awarded to Katalin Kariko and Drew Weissman for their contributions to nucleotide base modification and mRNA vaccines, which made it possible to develop mRNA vaccines against COVID-19 and significantly reduce the risk of COVID-19 infection in humans [86,87]. In summary, mRNA technology has certain advantages in the research and development of vaccines against fulminant infectious diseases, and mRNA vaccines are expected to become a new type of vaccine for preventing infectious diseases [88,89].

Kim and colleagues reported the efficacy of an mRNA vaccine in a mouse model of SFTS [90]. They proposed mRNA vaccine candidate genes encoding sGn-H or sGn-H-FT, both of which exhibit potent immunogenicity and protective effects. The authors intramuscularly injected 6- to 8-week-old BALB/c female mice with 1 μg of sGn-H mRNA lipid nanoparticles (LNPs) or sGn-H-FT mRNA LNPs at weeks 0 and 3, and blood samples were collected at weeks 0, 2, 5, 9, 12, and 15. The results showed that anti-sGn-H antibody titers remained high even at week 15, indicating a highly effective and durable immune response after immunization with sGn-H or sGn-H-FT mRNA LNPs. Crucially, A129 mice inoculated with 3 μg of sGn-H and sGn-H-FT mRNA LNPs were completely protected from the fatal challenge of DBV, while all control mice died. In conclusion, SGN-H and SGN-H-FT mRNA LNPs are promising vaccine candidates that provide protection against DBV infection. Nevertheless, further research is needed on how to extend the study period for antibody responses and viral challenges that will provide insight into the long-term duration and protective strength of SGN-H or SGN-H-FT mRNA vaccines.

Lu and colleagues recently reported the development of a DBV glycoprotein (including receptor structure and fusion structure domains) mRNA vaccine, which has been shown to confer type 1 helper T cells in 6- to 10-week-old A129 mice [91]. This cellular immune response can be achieved with a double dose at very low doses (1 μg), providing long-term and complete protection against DBV. This result is different from those of previous studies, which indicated that only a humoral response elicited by Gn/Gc or only a cellular response prompted by an NP/NS/LP combination can offer complete protection. The discrepancies between the aforementioned studies may be attributed to variations in vaccine reactivity among species and individuals. Further studies have demonstrated that the conserved epitopes of the full-length SFTSV glycoprotein, which are present in the conserved regions of the virus, promote a broad spectrum of protection against the virus through the activation of cellular immune responses. Caution is advised when using large proteins as antigens, given the current technical limitations in preparing oversized mRNAs and the potential presence of adverse epitopes that may influence the final outcomes. Furthermore, the utilization of animal models for efficacy assessments in preclinical studies may result in discrepancies in protection, as there is a paucity of both immunocompetent and cost-effective rodent models [92,93]. In essence, these findings demonstrate that the total length of SFTSV glycoprotein mRNA is optimal for DBV and other ribbon virus vaccine candidates. Furthermore, the use of conservative antigens provides guidance and a framework for the development of a broad-spectrum vaccine.

## Discussion

DBV was first discovered in 2009 in China. At present, the notification rate of SFTS has consistently increased, and the fatality rate is as high as 30%, posing a considerable threat to the public health system. Although DBV was previously listed as a priority pathogen by the WHO and many scholars have conducted extensive research on DBV, there remains no clinically approved vaccine. Protein subunit vaccines such as the FT nanoparticle vaccine have high efficacy and are well tolerated, exhibiting outstanding protection efficacy in mouse and aged ferret models. Nevertheless, the high production costs and necessity for multiple immunizations have limited their broader application. Furthermore, the optimization of immunization protocols is necessary to enhance vaccine-mediated immunity while minimizing reactogenicity. Live attenuated vaccines, such as rHB2912aaNSs and rHB29NSsP102A, have the potential to induce a robust humoral immune response and protect ferrets from lethal DBV challenge. However, safety considerations exist due to attenuated viruses having the potential risk of recovering their toxicity through genetic drift. Although the rHB29NSsP102A mutation is not detected in cell culture experiments, its genetic stability during in vivo passaging remains to be studied. At present, there is much research on recombinant virus vector vaccines. The safety and protective effects of these vaccines are relatively ideal in immunodeficient mouse models, but the specific mechanism of their cellular immune remains is still unclear and requires further clarification. DNA vaccine research and development technology is relatively mature and suitable for quickly responding to emerging pathogens. Nevertheless, DNA vaccines have certain limitations, such as a lack of immunogenicity, and the induced neutralizing antibody titers are not ideal [82,94]. mRNA vaccines can reduce production costs and induce high titers of neutralizing antibodies which persist for a long time. However, a comprehensive understanding of the potential long-term protective effects of mRNA vaccines warrants further extensive study. In addition, the effectiveness of these DBV candidate vaccines has only been validated in small animal models, and there is a lack of experimental data from nonhuman primates, and a paucity of clinical data.

In summary, although many studies have been conducted on DBV vaccine development, there remain still many challenges. The first step is to establish an appropriate animal infection model to evaluate the effectiveness of vaccines in vivo. To date, many rodent models of DBV have been developed, but no model fully reproduces human SFTS disease phenotypes [35]. A DBV infection model especially an optimal nonhuman primate model with a normal immune status is crucial for testing vaccine effectiveness. Second, a standard neutralization test method is needed to evaluate the cross-protection effect of the developed vaccines. Although some scholars have reported a detection method based on serum neutralizing antibodies [95], the cross-reactivity of these antibodies with different DBV genotypes still needs to be further evaluated to determine the standard strains for vaccine production.

## Summary

The occurrence of an SFTS epidemic necessitates the development of safe, effective, and affordable vaccines. To date, there are multiple promising candidates in the preclinical research stage for various types of vaccines, but no vaccine candidates have entered the clinical research stage. The research and development of marketing vaccines still faces many challenges in the future, including comprehensive evaluation of their effectiveness and safety in clinical application, cost issues, and vaccine storage and transportation.
